# Enhancing Biodegradation of Poly(lactic acid) in Compost at Room Temperature by Compounding Jade Particles

**DOI:** 10.3390/polym17152037

**Published:** 2025-07-26

**Authors:** Lilian Lin, Matthew Joe, Quang A. Dang, Heon E. Park

**Affiliations:** 1 Department of Chemical and Paper Engineering, Western Michigan University, Kalamazoo, MI 49008, USA; lilian.lin@wmich.edu (L.L.); matthew.k.joe@wmich.edu (M.J.); 2 New Zealand Institute for Minerals to Materials Research, Greymouth 7805, New Zealand; quang.anhdang@nzimmr.co.nz; 3 Department of Chemical and Process Engineering, University of Canterbury, Christchurch 8041, New Zealand

**Keywords:** poly(lactic acid), PLA, 3D printing, compression molding, biodegradable plastic, New Zealand jade, pounamu, enhanced biodegradation, tracking biodegradability, differential scanning calorimetry, degree of crystallization

## Abstract

Although PLA is an attractive biodegradable polymer, its degradation under natural conditions is often slow. This study investigates whether incorporating pounamu (New Zealand jade) particles into PLA can enhance its biodegradation rate under composting conditions at room temperature. PLA composites containing 0 to 15 wt% pounamu were fabricated using both compression molding and 3D printing. A simple, reproducible protocol based on residual mass measurement was developed to monitor the biodegradation process over a 12-month period. The results showed that increasing pounamu content consistently accelerated mass loss of the composite in the compost, indicating enhanced biodegradation. The 3D-printed samples degraded more rapidly than compression-molded ones. This was attributed to the layered structure, internal microcavities, and lower crystallinity of the 3D-printed samples, which provided greater surface area and accessibility for microbial activity. These findings highlight the dual role of pounamu as both a crystallization promoter and a facilitator of biodegradation and underscore the importance of the processing method when designing biodegradable polymer composites for real-world applications.

## 1. Introduction

The detrimental impact of plastic pollution on the global environment is well established and remains a pressing issue that demands immediate and coordinated mitigation efforts. The pervasive nature of this problem is highlighted by the detection of microplastics even in remote regions such as New Zealand [1], despite its geographical isolation. In response to the urgent need to combat plastic pollution, substantial research has focused on the development and use of biodegradable plastics [2]. These materials have attracted considerable attention as promising alternatives, offering the potential to reduce environmental harm due to their capacity to break down over time.

However, the widespread adoption of biodegradable plastics faces several significant challenges. Most notably, their degradation rate in natural environments is often slower than desired, raising concerns about their overall effectiveness in mitigating plastic accumulation. Additionally, compared to conventional plastics, biodegradable polymers often exhibit inferior mechanical properties, limiting their practical use across various industries. These limitations have contributed to the cautious and gradual integration of biodegradable plastics into mainstream applications. In this study, we aim to address the issue of suboptimal biodegradation of poly(lactic acid) (PLA) in natural conditions through the compounding of jade particles, wherein we utilize a facile approach to monitor this biodegradation over a 12-month period.

### 1.1. Challenges with PLA

Poly(lactic acid) (PLA) is one of the most widely used synthetic, bio-based plastics in both industry and research, with applications spanning three-dimensional (3D) printing [3,4,5], fabrics, packaging, bottles, films, automotive components, electronics, and tissue engineering [6]. Certain PLA grades exhibit properties comparable to those of conventional, non-degradable plastics. More than 200,000 patents have been published on PLA-related technologies [7], and the number of academic publications continues to grow exponentially, covering topics such as phase and stereocomplex behavior [8,9,10,11], rheology [12,13,14,15,16], foaming [16,17,18,19,20,21], recycling [3,16,22,23,24], and composite developments [5,25,26,27]. Numerous reviews and monographs further underscore the extensive research interest in this material [6,7,28,29,30,31,32,33,34,35,36,37,38,39,40].

Despite being marketed as “biodegradable” [41,42,43], PLA degrades very slowly under ambient and even industrial conditions. Additionally, in soil, freshwater, or marine environments, it often takes years or even decades to break down significantly [42,44]. This slow degradation is primarily due to the glass transition temperature (approximately 55–60 °C) of PLA, which is higher than typical ambient temperatures, and to its inherent hydrophobicity and semi-crystalline structure, both of which hinder water absorption and microbial enzymatic activity. Studies have reported minimal mass loss or carbon dioxide ($CO_{2}$) evolution from PLA exposed to soil and water under ambient conditions, even over extended periods [44]. This disparity between perceived and actual degradation performance, along with the need for specialized industrial composting facilities, which are not universally available, poses major challenges for the end-of-life management of PLA [45]. Moreover, it is unrealistic to expect that PLA will degrade naturally in marine environments or soil at elevated temperatures like 60 °C or that microorganisms capable of such degradation could thrive under those conditions. This undermines the very purpose of using biodegradable plastics, which are intended to break down naturally when released into the environment.

Thus, it is crucial to develop effective strategies to enhance the degradability of PLA at mild temperatures, ideally around 30 °C, where microbial activity is more relevant to real-world conditions [44,46]. However, modifications designed to improve degradability often come at the expense of mechanical strength or processability, resulting in trade-offs that limit the applicability of PLA [27,47]. There remains a strong need for approaches that can accelerate PLA degradation without significantly compromising or improving its functional performance.

### 1.2. Methods to Enhance Biodegradation Speed

To address the issue of PLA’s slow biodegradation, a variety of strategies have been explored to tailor its degradation rate [48]. These approaches include physical, thermal, mechanical, radiation-based, chemical, and biological pretreatments, as well as compounding with other materials. Physical treatments [49] aim to alter the structural properties of PLA, making it more susceptible to biological degradation. Thermal treatments such as annealing or controlled heat exposure can modify the degree of crystallinity, which in turn affects water penetration and enzymatic accessibility [50]. Mechanical treatments, including milling and grinding, increase the surface area of PLA, thereby enhancing interaction with enzymes and microorganisms [51]. Radiation-based techniques, particularly UV irradiation, can induce photodegradation, reducing the molecular weight of PLA and potentially enhancing its biodegradability [52]. Chemical modifications are also widely used to accelerate PLA degradation. Hydrolytic pretreatment, involving exposure to moisture or water, especially at elevated temperatures, can initiate cleavage of ester bonds in the backbone of PLA [53,54,55]. Enzymatic hydrolysis under mild conditions can further modify the surface, increasing hydrophilicity and exposing functional groups for microbial attack [56,57,58]. Oxidative pretreatments, such as advanced oxidation processes (AOPs) [56], can promote surface oxidation, facilitating microbial colonization. Specific additives, like phosphites, have been shown to accelerate hydrolysis [54]. Biostimulants such as gelatin, skim milk, and ethyl lactate can be added to compost matrices to enhance protease activity and stimulate specific microorganisms, thereby improving PLA biodegradation under mesophilic conditions [57]. Inorganic additives like iron compounds, e.g., ${Fe}_{3}O_{4}$ nanopowder, have also demonstrated the ability to accelerate PLA degradation, possibly by catalyzing hydrolysis reactions, especially when used alongside biostimulants at ambient temperatures [57,58,59]. Although many of the above treatments improve degradability, they often compromise the mechanical properties of PLA, which is already relatively weak compared to conventional plastics. As a result, strategies that simultaneously enhance both biodegradation and mechanical performance have been investigated. These include compounding PLA with more readily biodegradable polymers [60], with natural or synthetic polymers that improve water uptake and microbial accessibility [27,61,62,63,64,65], or with hydrophilic fillers and particles that increase surface area and permeability to water, thereby facilitating hydrolytic degradation [66,67].

### 1.3. Methods to Monitor Biodegradation

Understanding the biodegradation kinetics of biodegradable polymers including PLA is critical for assessing its environmental impact and informing material design. Biodegradation is a complex, multi-stage process, including biodeterioration, fragmentation, assimilation, and mineralization, affected by environmental conditions, microbial activity, and polymer properties [68]. To standardize biodegradability assessments, international protocols have been developed. Key examples among them [69] are ISO 14855 [70] and ASTM D5338 [64], which measure the aerobic biodegradation of plastics under controlled composting conditions at thermophilic temperatures by tracking $CO_{2}$ evolution. The percentage of biodegradation is calculated based on the $CO_{2}$ released compared to the theoretical value for complete mineralization and benchmarked against a cellulose control. Other standards, such as ISO 20200 [71] and ISO 21701 [72], address compostability in soil–compost mixtures and hydrolytic degradation, respectively. These frameworks are essential for regulatory compliance and product labeling. Various analytical techniques [57,58,73,74] are used to monitor biodegradation. Respirometry tracks oxygen consumption or $CO_{2}$ production in aerobic systems [57,58,75], while FTIR and GPC assess chemical structure changes and molecular weight reduction, respectively. Additional methods like clear-zone assays and conductivity measurements offer insights into microbial activity. However, these techniques often require expensive instrumentation and maintenance, potentially limiting broader research efforts.

As a simpler alternative, mass loss measurements provide a practical and cost-effective way to track degradation by comparing the material’s initial and final weights [76,77]. Developing such accessible methods can help accelerate biodegradability studies and broaden participation in this field.

### 1.4. New Approach

To address the dual limitations of PLA, slow degradation and low mechanical strength, our previous study [27] explored the incorporation of New Zealand jade (pounamu) particles into PLA. Pounamu, also known as nephrite jade or greenstone, is a tough silicate mineral composed primarily of actinolite or tremolite. It holds cultural significance for the Māori people and is widely used in carving. During the carving process, 20–30% of the jade is typically lost as fine or coarse waste particles, which are often discarded. Recognizing the potential of this waste material as a sustainable additive, we previously demonstrated that incorporating pounamu microparticles into PLA significantly accelerated thermal degradation, as evidenced by thermogravimetric analysis (TGA) and rheological time sweep tests, while also enhancing mechanical strength [27]. These results suggested that pounamu may exert a catalytic or destabilizing effect on the ester bonds of PLA at elevated temperatures, while simultaneously reinforcing the polymer matrix. Building on these findings, we hypothesize that pounamu may also influence hydrolytic biodegradation, a key mechanism in composting environments. Possible mechanisms include enhanced water absorption due to the particles’ hydrophilic properties, formation of interfacial pathways that facilitate hydrolysis, or alterations in PLA crystallinity or morphology that promote microbial activity. As a mineral filler, pounamu could also contribute to increased stiffness and strength, offering a dual benefit of mechanical reinforcement and enhanced biodegradation.

**Figure 1:**
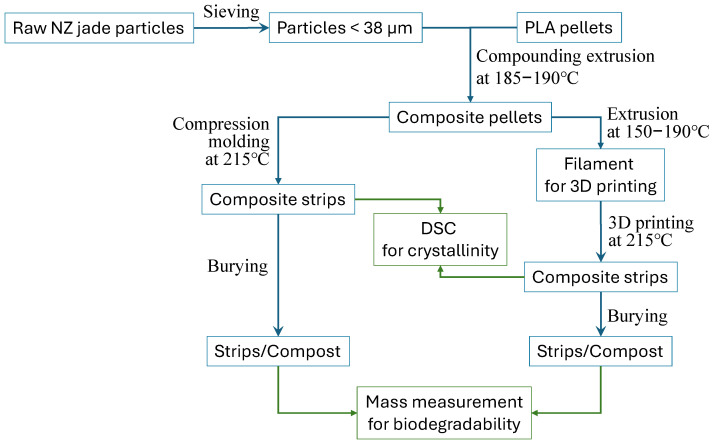
Workflow diagram. Blue arrows indicate process while green arrows indicate measurement.

The aim of this research is exploring the potential of pounamu particles as a dual-function additive to enhance both biodegradation and mechanical strength of PLA composites in composting environments. To achieve our aim, we tested this hypothesis by investigating the effect of incorporating 0–15 wt% pounamu particles into PLA on its biodegradation rate under mild, controlled composting conditions. Over a 12-month period, biodegradation was primarily assessed by monitoring mass loss using a simple, accessible measurement protocol that we developed in this study. We also compare two processing methods, compression molding and 3D printing, to evaluate how manufacturing technique influences degradation behavior. The overall workflow is shown in Figure 1. The significance of this study is to provide a compelling approach to addressing the slow degradation rate of PLA under natural conditions, which is a major limitation in its broader adoption as a biodegradable alternative to traditional plastics. Along with our previous study [27], the outcome of this study reveals that incorporating pounamu (New Zealand jade) particles can induce a synergistic effect that not only enhances biodegradation but also reinforces mechanical properties.

## 2. Materials and Methods

### 2.1. Materials

Poly(lactic acid) (PLA) was selected as the base polymer matrix due to its biodegradability and its ability to produce final products with reasonable dimensional precision [78]. For this study, a commercial PLA grade (Ingeo™ 2003D, NatureWorks LLC, Blair, NE, USA) was used. This PLA has a melt flow index of 3.2 g/10 min (measured at 190 °C under a 2.16 kg load), a D-lactide content of 4.3% [79], and a weight-average molecular weight (Mw) of approximately 230,000 g/mol [80]. Compost used for the biodegradation study was Tui Compost Essentials (Tui Garden Products, Mount Maunganui, New Zealand) containing organic matter, blood, bone, and gypsum, purchased from a local hardware store (Mitre10, Christchurch, New Zealand). Pounamu (nephrite jade) [81], sourced from the Waewae Pounamu shop in Hokitika, New Zealand, was used as the reinforcing and degradation-accelerating additive. These jade particles, a byproduct of carving processes, were incorporated into the PLA matrix to form composite samples. The chemical structure and physical properties can be found in our previous publication.

### 2.2. Methods

#### 2.2.1. Screening NZ Jade Particles

To refine the particle size distribution of pounamu, a sieve process was conducted. The raw jade particles, obtained from the supplier as ground material, were first dried in a vacuum oven (Model 8100, Contherm Scientific, Lower Hutt, New Zealand) at 105 °C for 24 h to remove residual moisture. After drying, the particles were subjected to sieving using an electromagnetic sieve shaker (EML 200 Premium, Haver & Boecker, Oelde, Germany). A series of sieves with decreasing mesh sizes, 250, 212, 106, 75, and 38 µm, were stacked from top to bottom. The shaker used vibration to separate particles based on size. The weight of particles collected between two sieves was measured. As shown in Table 1, the majority of raw particles provided by the supplier were already 38 µm or smaller, making them suitable for consistent dispersion within the PLA matrix. For this study, only the fraction collected from the 38 µm sieve was selected for use in composite fabrication.

#### 2.2.2. Composite Preparation (Extrusion Compounding)

The raw PLA pellets were dried in a vacuum oven at 45 °C overnight to remove moisture and residual volatiles prior to extrusion compounding. Four PLA/pounamu composites were prepared, as summarized in Table 2, using a twin-screw extruder (Labtech Engineering Co. Ltd., Samutprakarn, Thailand) equipped with a 2 mm, 3-hole die. The extrusion conditions are detailed in Table 3. Initially, the PLA pellets and pounamu particles were manually premixed and then fed into the extruder hopper. After extrusion, the extruded strands were cooled in a water bath and pelletized to produce cylindrical composite pellets. These pellets were then dried again in the vacuum oven at 45 °C overnight to ensure thorough removal of any residual moisture. The resulting dried composite pellets were used as feedstock for filament fabrication (eventually for 3D printing) or for producing strips via compression molding.

#### 2.2.3. Fabricating Filament for 3D Printing

The pellets of each composite were individually processed using the Labtech twin-screw extruder equipped with a 2 mm, single-hole die under the conditions specified in Table 4. The extruded filaments were cooled in a water bath and subsequently wound onto spools designed for 3D printing. The target filament diameter was 1.75 mm, compatible with standard 3D printers. To ensure optimal printing quality and minimize moisture-related defects, the spools were dried in a vacuum oven at 45 °C for at least 24 h prior to 3D printing.

#### 2.2.4. Three-Dimensional-Printing Strips

Composite strips were fabricated using an M2 3D printer (MakerGear, Ltd., Beachwood, OH, USA), equipped with a 0.75 mm nozzle, utilizing filaments from each composite. The printing parameters were set to an extrusion temperature of 215 °C and a bed temperature of 55 °C. The strips were printed with a layer thickness of 0.2 mm, 100% infill, and a raster orientation of −45°/45°. Each strip measured 35 mm × 12.7 mm × 3.2 mm, with an approximate mass of 1.7 g. To ensure consistent material properties, all printed strips were stored in a vacuum oven (OV11, Jeio Tech, Daejeon, South Korea) at room temperature for at least one week prior to testing.

#### 2.2.5. Compression-Molding Strips

After drying the composite pellets in a vacuum chamber (OV11, Jeio Tech, Daejeon, South Korea) at room temperature for over one week, they were reprocessed by compression molding to fabricate composite sheets. Each composite batch was compression-molded individually using a hot press (Model 3912, Carver, Inc., Wabash, IN, USA) with a 125 mm × 125 mm × 3 mm mold. The molding was carried out under a force of 5 tons at 215 °C. Upon completion, the hot press was rapidly cooled with water to minimize thermal degradation. Once the mold temperature dropped below 30 °C, the composite sheet was released and cut into strips measuring 35 mm × 12 mm × 3 mm, with an approximate mass of 1.5 g. All molded strips were subsequently stored in a vacuum oven at room temperature for at least one week prior to testing.

#### 2.2.6. Moisture Content of Compost

The moisture content of the compost was measured using an MA35 infrared moisture analyzer (Sartorius AG, Göttingen, Germany). Approximately 5 g of compost was evenly spread on the sample pan of the analyzer. The instrument operated in automatic mode with a drying temperature set to 105 °C, a standard condition for determining moisture in organic materials. Drying continued until the rate of mass loss fell below the instrument’s threshold, indicating completion. Moisture content was calculated as the percentage of mass lost relative to the initial sample weight. Each sample was tested in triplicate to ensure reproducibility, resulting in an average moisture content of 22.5 ± 2.1 wt%.

#### 2.2.7. Degree of Crystallinity

The degree of crystallinity of the eight composite strips was determined using a thermal analyzer (STA8000, Perkin-Elmer, Waltham, MA, USA) operating in differential scanning calorimetry (DSC) mode. Small pieces (10–20 mg) were cut from each composite strip and loaded into the instrument. Heat flow was recorded from 30 °C to 200 °C at a heating rate of 10 °C/min. The melting peak, observed near 160 °C, was integrated to determine the heat of fusion. The degree of crystallinity ($X_{C}$) was calculated using the following equation:

(1) $$X_{C}=\frac{\Delta H_{m}/\phi }{\Delta H_{m}^{0}}\times 100\%$$

where $\Delta H_{m}$ is the heat of fusion (J/g) of the composite, $\Delta H_{m}^{0}$ is the heat of fusion of a 100% crystalline form of PLA, which is 93 J/g [82], and ϕ is the mass fraction of PLA in the composite, which is necessary to exclude the non-melting pounamu content. $\Delta H_{m}$ was determined as the area under the DSC curve as shown in Figure S1 in the Supplementary Materials.

#### 2.2.8. Placing Samples in Compost and Mass Measurements

**Figure 2:**
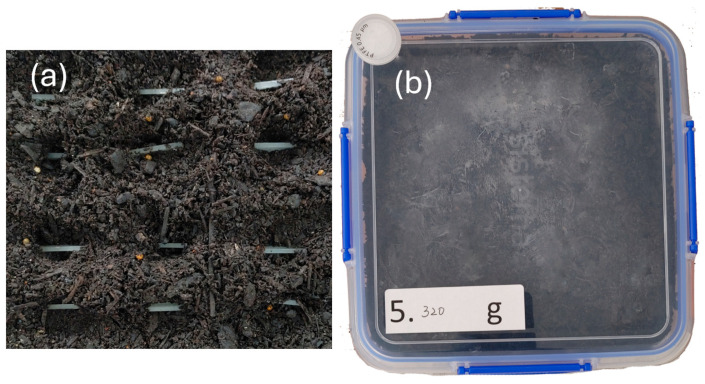
(**a**) Sample strips half-buried in compost (Top left: sample to be taken out at Month 1, Top right: Month 3, Bottom right: Month 12). There were seven more containers with sample strips prepared by compression molding or 3D printing with various pounamu contents. (**b**) Sample container after closing the cover. The mass of the container is 320 g. The filter is shown in the left top corner.

Thirteen strips were prepared for each of the eight sample types (two processing methods × four pounamu contents), resulting in a total of 104 strips. Prior to composting, all strips were cleaned with soapy water, rinsed with deionized water, dried using Kimwipes^®^ (Kimtech Science brand™, Kimberly-Clark Professional, Irving, TX, USA), and placed in a vacuum oven (OV11, Jeio Tech, Daejeon, South Korea) at 25 °C with a −40 °C cold trap for 24 h. The initial mass ($M_{i}$) of each strip was measured at 25 °C using a microbalance (Sartorius CPA124S, Göttingen, Germany). Eight sets of 12 strips (one set per sample type) were buried in eight separate sealed plastic containers, each filled with 1.5 kg of compost. As shown in Figure 2a, the strips were placed vertically (with the broad surface perpendicular to the ground) to reduce gravitational effects. Then 0.5 kg of compost was added and packed over the strips to ensure full coverage. Each container was fitted with a PTFE membrane syringe filter (0.45 μm pore size, 25 mm diameter, STF grade, CHMLab, Terrassa, Spain) on the lid (Figure 2b) to allow aeration while minimizing moisture loss.

At the end of each month (Months 1–12), one strip was retrieved from each compost container (Month 0 samples were not buried but used to record initial mass). Retrieved strips were gently washed with soapy water, rinsed with deionized water, dried with Kimwipes^®^ (Kimtech Science brand™, Kimberly-Clark Professional, Irving, TX, USA), and placed in a vacuum oven (OV11, Jeio Tech, Daejeon, South Korea) at 20 °C with a −40 °C cold trap for 24 h. This drying step was assumed not to affect degradation outcomes. The mass of each strip was then measured using the same microbalance. To maintain consistent compost conditions, any loss from the original 2 kg was compensated by spraying deionized water as needed, though compost mass loss was minimal throughout the study. The residual mass percentage of polymer, used to evaluate biodegradation kinetics, was calculated as:

(2) $$M_{R}\;\left(t\right)\;\left(\%\right)=\frac{{\phi M}_{i}-[M_{i}-M\left(t\right)]}{{\phi M}_{i}}\times 100\%$$

where $M_{i}$ is the initial mass of a sample strip (Month 0) before composting and $M\left(t\right)$ is the mass of the same strip at the end of each month (from Month 1 through Month 12). These two quantities correspond to the same sample. The term *ϕ* represents the mass fraction of PLA in the composite strip, used to account for the presence of non-degradable pounamu filler. Thus, ${\phi M}_{i}$ is the initial mass of the pure polymer portion in the composite strip, $M_{i}-M\left(t\right)$ is the mass loss of the pure polymer, and ${\phi M}_{i}-[M_{i}-M\left(t\right)]$ is the residual mass of the pure polymer in the composite strip. To avoid potential artifacts introduced by repeated handling, no samples were returned to the compost after mass measurement. Each monthly measurement was conducted on a fresh sample strip that had undergone only one mass measurement at Month 0.

Before each mass measurement, the microbalance (Sartorius CPA124S, Göttingen, Germany) was recalibrated using its internal isoCAL function to ensure measurement accuracy. While biodegradation likely occurs primarily at the surface, no normalization to surface area was applied, as all strips had consistent dimensions and mass, ensuring comparable surface-to-mass ratios across all samples.

## 3. Results and Discussion

**Figure 3:**
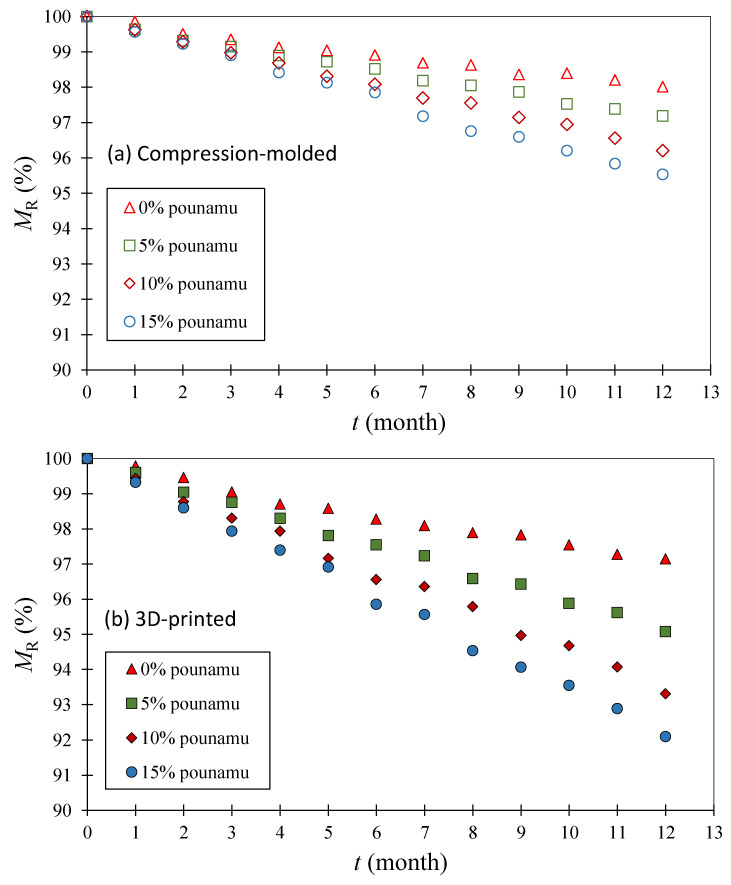
Residual mass in compost after each month. (**a**) compressed-molded samples, (**b**) 3D-printed samples.

Figure 3 shows the residual mass $M_{R}\;\left(t\right)$ over 12 months for all sample types, categorized by the shaping process and pounamu content. Overall, the degradation was relatively limited, with the highest mass loss reaching only 8% in the case of 3D-printed samples containing 15 wt% pounamu. This degradation extent is notably lower than in previous studies [67,83,84], which report nearly complete biodegradation of PLA-based materials within a few months. However, those studies primarily utilized thin films (<1 mm thickness) or finely divided particles (<1 mm diameter), enabling degradation to proceed in a three-dimensional manner due to the increased surface area. In contrast, our samples were significantly thicker (>3 mm), limiting degradation to primarily one-dimensional surface attack. For example, a study [65], which followed ISO 14855-2 [85] protocols (28 °C) evaluated biodegradation of PLA blended with more degradable polymers via CO_2_ evolution and showed at least 40% degradation within one year when PLA was compounded with 20 wt% or more of biodegradable additives. Since cellulose (used as the reference standard in that method) degrades almost completely under similar conditions [86], the reported values may be interpreted as near-absolute biodegradation percentages. In comparison, our study observed substantially lower biodegradation, likely due to differences in sample geometry as mentioned earlier. ISO 14855-2 recommends particulate solid samples (<250 µm), which offer much higher surface-to-mass ratios and microbial accessibility. Our bulk strip samples, in contrast, were designed to simulate more realistic usage scenarios, sacrificing biodegradation rate for practical relevance.

Additionally, our samples were composted under relatively mild temperatures, which is a critical factor influencing PLA degradation rates [87]. Mayekar and Auras [58] reported that the molecular weight of PLA can decrease by 50% in six months in the presence of biostimulants. However, as discussed by Lin et al. [27], molecular weight loss reflects chain scission, i.e., partial degradation, while mass loss corresponds to complete decomposition. Therefore, it is possible that significant molecular degradation (chain scission) or fragmentation occurred in our samples without being fully reflected in the mass loss measurements, as long as those portions remain solid. On the other hand, this mass loss confirms that solid plastic has transformed into gas molecules, which we assume is due to biodegradation. Thus, it should also be noted that the complete degradation was propagated from the sample surface without generating fragments or microplastics based on the observation. At a glance, the sample mass could increase upon chain scission. Considering PLA is polymerized by condensation, i.e., removing water molecules from monomers, it is tempting to imagine the reverse of condensation polymerization (i.e., chain scission) would just plug water back into the chain. However, when it comes to the actual hydrolytic degradation (chain scission) of PLA, the mass increase is not quite what we would expect. Water reacts with the ester linkages in PLA and cleaves the chains, forming shorter chains, but the amount of water added is small relative to the polymer’s mass. Thus, although there is a chemical addition of water, the overall mass gain should be negligible, not something we can observe on a balance. Then again, this was not the case based on the observation in this study.

**Figure 4:**
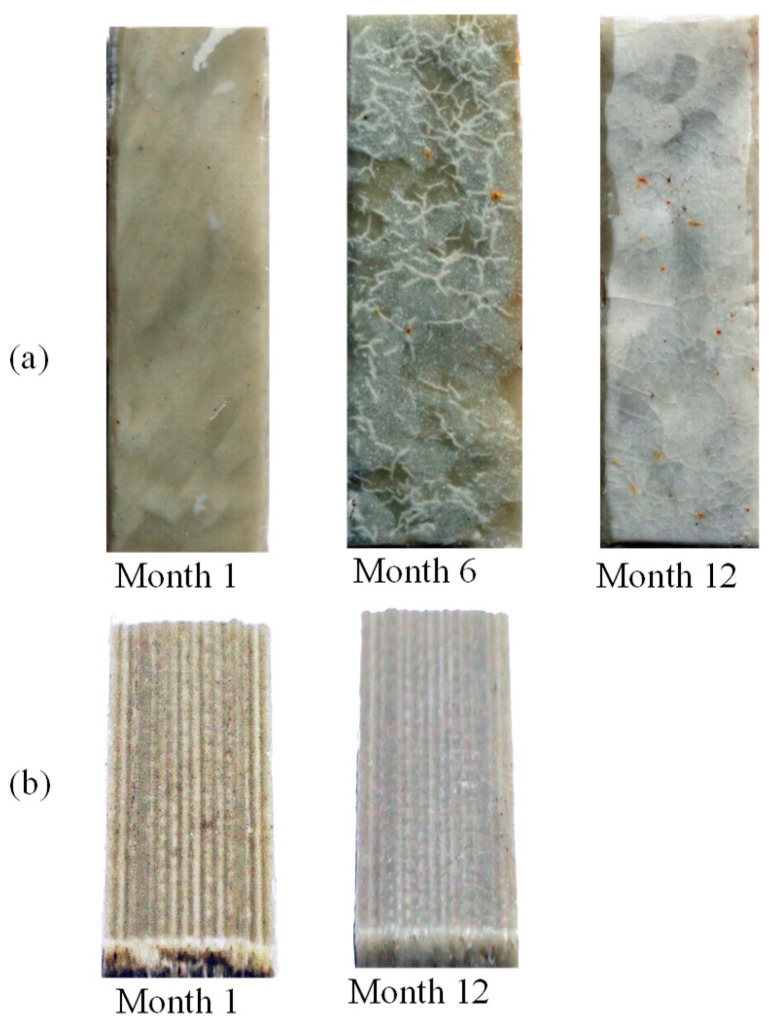
Surface of samples after treating in compost. (**a**) Top view of compression-molded sample with 15 wt% pounamu, (**b**) 45-degree view (to elaborate the grooves) of 3D-printed sample with 15 wt% of pounamu.

Figure 3 also shows that, among our samples, 3D-printed strips consistently degraded faster than compression-molded ones. This can be attributed to the characteristic layered structure of fused filament fabrication, which results in surface grooves and imperfections. These features increase surface area and possibly enhance oxygen and moisture penetration [88], both of which accelerate microbial activity and hydrolysis. Although surface grooves were visually smoothened during degradation (Figure 4b), their initial presence likely promoted early-stage biodegradation. Finally, increasing pounamu content clearly correlated with faster mass loss. This supports our hypothesis that pounamu particles may facilitate hydrolysis, possibly by increasing water uptake, disrupting PLA crystallinity, or providing catalytic mineral surfaces. In all cases, 3D-printed samples with higher pounamu content exhibited the most pronounced degradation.

We did not aim to develop detailed biodegradation kinetic models in this study. Rather, our objective was to demonstrate that incorporating pounamu particles can accelerate the biodegradation of PLA in compost at room temperature using a relatively simple quantitative approach, i.e., monitoring mass loss over time. While this method provides a practical indication of degradation behavior, more precise kinetic modeling would require advanced analytical techniques such as CO_2_ evolution measurements and molecular weight analysis to accurately characterize degradation pathways and rates.

The shaping process resulted in markedly different visual changes during biodegradation. As shown in Figure 4a, the compression-molded samples containing 15 wt% pounamu developed localized whitening over time. By Month 1, a small, whitened region appeared near the top edge, which became significantly more pronounced by Month 6, where network-like white lines emerged. These lines likely correspond to interfaces between individual pellets that were not fully fused during compression molding. Such interfaces may entrap air and form weak points in the matrix, making them more susceptible to microbial attack. In addition to these lines, small white and brown spots were observed. Previous studies have linked such color changes to microbial activity during biodegradation. Luo et al. [67] reported that brown discoloration in PLA under composting conditions indicates microbial colonization, while surface cracks suggest active degradation. Similarly, PLA has been reported to turn opaque white due to surface fragmentation in both compost [89] and landfill conditions [90]. These changes in appearance thus reflect progressive physical and chemical deterioration of the material. While these effects are most visible at the surface, they likely propagate slowly inward, indicating that degradation is not confined to a superficial layer. By Month 12, the compression-molded sample exhibited widespread whitening across its surface, along with a few brown spots, suggesting more advanced biodegradation.

In contrast, the 3D-printed samples exhibited a more uniform and predictable pattern of morphological change (Figure 4b). Owing to the layer-by-layer nature of fused filament fabrication, the printed strips initially displayed visible grooves, particularly on the upper surface. These grooves, caused by incomplete interlayer fusion, were easily perceptible by touch and appeared greenish due to the color of the embedded pounamu particles. By Month 1, white parallel lines began to emerge along these groove patterns, indicating early-stage localized degradation. Over time, the overall color of the strips shifted to a grayish tone, becoming similar in appearance to the compression-molded samples by Month 12. It appears that the elevated surface roughness and groove structure of the 3D-printed samples facilitated microbial attachment and localized enzymatic attack. The raised ridges and lines may have acted as initiation sites for crazing and crack formation, which then propagated inward with time. This layered morphology, unique to the 3D-printing process, likely contributed to the accelerated and more uniform degradation observed in printed samples relative to their compression-molded counterparts although 3D-printed products are weaker than compression-molded counterparts [27].

**Figure 5:**
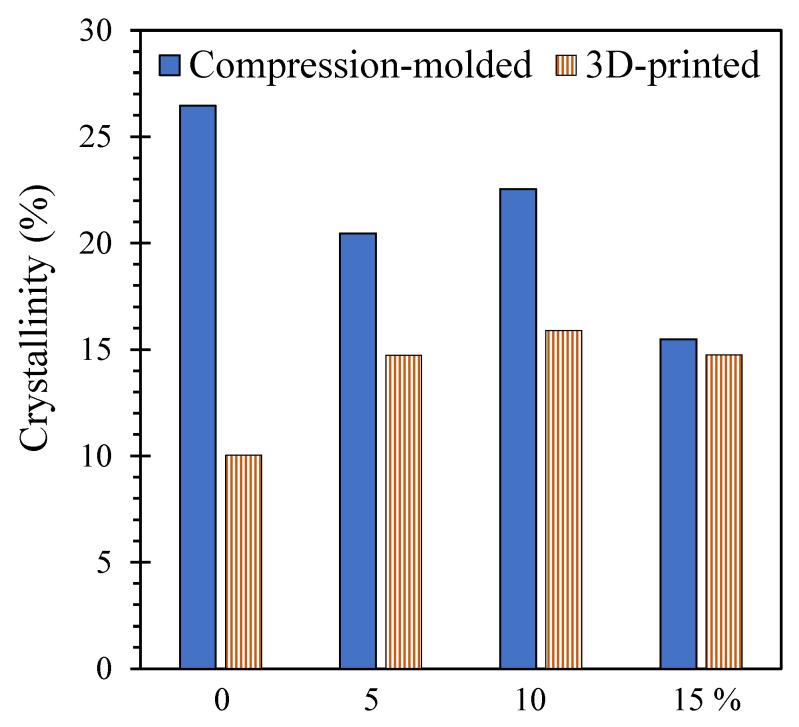
Degree of crystallinity of PLA/pounamu composites. X-axis shows the pounamu content in wt%.

Figure 5 presents the degree of crystallinity of the composites at Month 0. The compression-molded strips were rapidly cooled (quenched) after pressing to minimize thermal degradation during the shaping process, which likely limited the time available for polymer chains to organize into crystalline structures. In contrast, 3D printing allows for slower cooling during extrusion, which can facilitate more crystalline growth. However, compression-molded samples exhibit higher crystallinity than their 3D-printed counterparts in Figure 5. This difference can be attributed to the effects of pressure and annealing time during compression molding. Specifically, the high pressure and extended thermal exposure during compression molding allow polymer chains to pack more closely together, promoting the formation of crystalline regions [91]. For the 3D-printed composites, crystallinity increased with pounamu content as the fine jade particles acted as nucleating agents, facilitating crystallization. The presence of more nucleation sites enables the formation of crystalline regions during cooling, resulting in higher overall crystallinity. This effect is particularly pronounced when the polymer matrix is loosely connected as in 3D printing. In contrast, crystallinity decreased with pounamu content in compression-molded composites. In these samples, the polymer chains are already closely packed due to the applied pressure, and the irregular solid surfaces of the filler particles may act as impurities, disrupting the crystalline structure.

The effect of increasing crystallinity with pounamu content (for 3D-printed composite) presents a trade-off in the performance of biodegradable polymer composites. On one hand, as shown in our previous study [27], both higher crystallinity and the presence of rigid filler particles contribute to improved mechanical strength, an important advantage for practical use. On the other hand, higher crystallinity may slow biodegradation, especially in compression-molded samples (for compression-molded composite) as crystalline regions are less permeable to water and more resistant to enzymatic attack than amorphous regions.

However, the pounamu particles may still enhance biodegradation by providing additional surface area for microbial colonization, particularly in the more porous 3D-printed composites. A similar duality is observed when comparing compression-molded and 3D-printed samples. Despite having higher crystallinity, 3D-printed samples degraded faster (Figure 3). This behavior can be explained by their internal structure. In our previous study [27], compression-molded samples exhibited approximately twice the tensile strength and up to ten times the Young’s modulus of 3D-printed samples, indicating a denser and stiffer structure. These differences arise from the uniform pressure applied during compression molding, which thoroughly fuses the polymer melt. In contrast, 3D printing, with its layer-by-layer deposition, results in microcavities and weak interlayer bonding. Consequently, the more porous and flexible structure of the 3D-printed composites allows for greater microbial access and internal diffusion pathways, which can accelerate biodegradation despite higher crystallinity. Therefore, when designing biodegradable products, it is important to consider how the shaping process affects both mechanical properties and degradation behavior.

The selected PLA grade has been widely utilized in fresh food packaging (e.g., dairy containers, transparent clamshells, hinged containers, and cold drink cups), food serviceware (e.g., disposable cutlery, plates, and trays), filament for 3D printing, and extruded roll stock for thermoforming into various shapes. In light of these uses, the findings of this study can be applied to this variety of potential applications, particularly with enhanced mechanical strength and biodegradability. Additionally, other PLA grades may be considered as matrix materials incorporating pounamu particles for other applications.

## 4. Conclusions

This study developed a straightforward and accessible method to assess the biodegradation of PLA-based composites by monitoring mass loss in composting conditions over a 12-month period. Unlike more complex analytical approaches based on CO_2_ evolution or molecular weight analysis, our method offers a practical means to quantify the long-term degradation behavior of thick polymer samples in conditions that simulate real-world applications.

We demonstrated that the incorporation of pounamu (New Zealand jade) particles into PLA matrices enhanced the rate of biodegradation. Composites with higher pounamu content exhibited over 3 times greater mass loss (up to 8% loss under real-world applications), indicating that the particles may serve dual roles, acting as crystallization nucleation sites resulting in higher mechanical strength and providing surfaces that facilitate microbial enzymatic activity. Opposite effects on crystallization were observed with increasing pounamu content depending on the shaping method, with compression-molded and 3D-printed samples experiencing a −43% decrease and +40% increase in crystallinity, respectively. Although higher crystallinity is generally expected to hinder biodegradation by limiting water absorption and enzymatic access, our results suggest that the surface effects of pounamu particles, such as enhanced microbial colonization and enzymatic attack, offset these limitations, resulting in accelerated degradation, especially for 3D-printed composite. We also highlighted the significant influence of the shaping method on biodegradation behavior. Compression-molded and 3D-printed strips, although made from the same composite materials, showed distinct morphological changes and degradation profiles. The 3D-printed samples degraded more rapidly than compression-molded ones, likely due to their layered structure and greater surface roughness, which enhanced microbial access. This finding underscores the importance of considering fabrication methods when designing biodegradable products, as the physical structure, especially surface characteristics and internal porosity, can have a substantial impact on degradation rates.

Overall, this work provides valuable insight into how filler content and processing methods influence the degradation behavior of PLA composites. It also offers a simple yet effective strategy for tracking biodegradation in larger, application-relevant samples, helping bridge the gap between laboratory studies and real-world performance of compostable materials.

## Figures and Tables

**Table 1:** Particle size distribution after screening raw jade particles.

Screen Size (µm)	wt%
>212	0.12
106–212	0.30
75–106	1.09
38–75	2.46
0–38	96.04

**Table 2:** Composite formulation for extrusion compounding.

Code	PLA (wt%)	NZ Jade Particles (wt%)
0 wt%	100.0	0.0
5 wt%	95.0	5.0
10 wt%	90.0	10.0
15 wt%	85.0	15.0

**Table 3:** Conditions for extrusion compounding.

Temperatures (Die to Feed) (°C)	185–190
Screw speed (rpm)	180
Feed speed (rpm)	25–30 depending on the formulation
Pelletizer speed (m/min)	17.5
Pellet diameter (mm)	2.5
Running pressure (bar)	30–45 depending on the formulation

**Table 4:** Conditions for filament production.

Temperatures (Die to Feed) (°C)	150–190
Screw speed (rpm)	150
Feed speed (rpm)	20–30 depending on the formulation
Caterpillar speed (m/min)	25
Running pressure (bar)	50–70 depending on the formulation

## Data Availability

The original contributions presented in this study are included in the article/supplementary materials. Further inquiries can be directed to the corresponding author.

## Supplementary Materials

The following supporting information can be downloaded at: https://www.mdpi.com/article/10.3390/polym17152037/s1, Figure S1: DSC chromatogram. The area under the curve of the peak was used to calculate the ΔH_m_, the heat of fusion (J/g), which is based on the area under the curve of heat flow versus time and obtained by the operating software, Pyris 13 (Perkin-Elmer, USA). An example is shown for compression-molded 0 wt% sample. Data were vertically shifted to avoid confusion. Each division on Y-axis is 0.1 W/g. (a) compression-molded samples, (b) 3-D printed samples.
