# Supplementary material for: Enhancing Biodegradation of Poly(lactic acid) in Compost at Room Temperature by Compounding Jade Particles
Source: Polymers (Basel). 2025 Jul 26;17(15):2037. doi: 10.3390/polym17152037 (PMC12349505; doi:10.3390/polym17152037)
Supplement: Supplementary file 1 [file polymers-17-02037-s001.zip › polymers-3746531-supplementary.pdf]

Supplements

Enhancing Biodegradation of Poly(lactic acid) in Compost at Room Temperature by Compounding Jade Particles

Lilian Lin [1], Matthew Joe [1], Quang A. Dang [2,3] and Heon E. Park [1,*]

[1] Department of Chemical and Paper Engineering, Western Michigan University, Kalamazoo, MI 49008, USA; lilian.lin@wmich.edu (L.L.); matthew.k.joe@wmich.edu (M.J.)
[2] New Zealand Institute for Minerals to Materials Research, Greymouth 7805, New Zealand; quang.anhdang@nzimmr.co.nz
[3] Department of Chemical and Process Engineering, University of Canterbury, Christchurch 8041, New Zealand
* Correspondence: heon.park@wmich.edu; Tel.: +1-269-276-3508

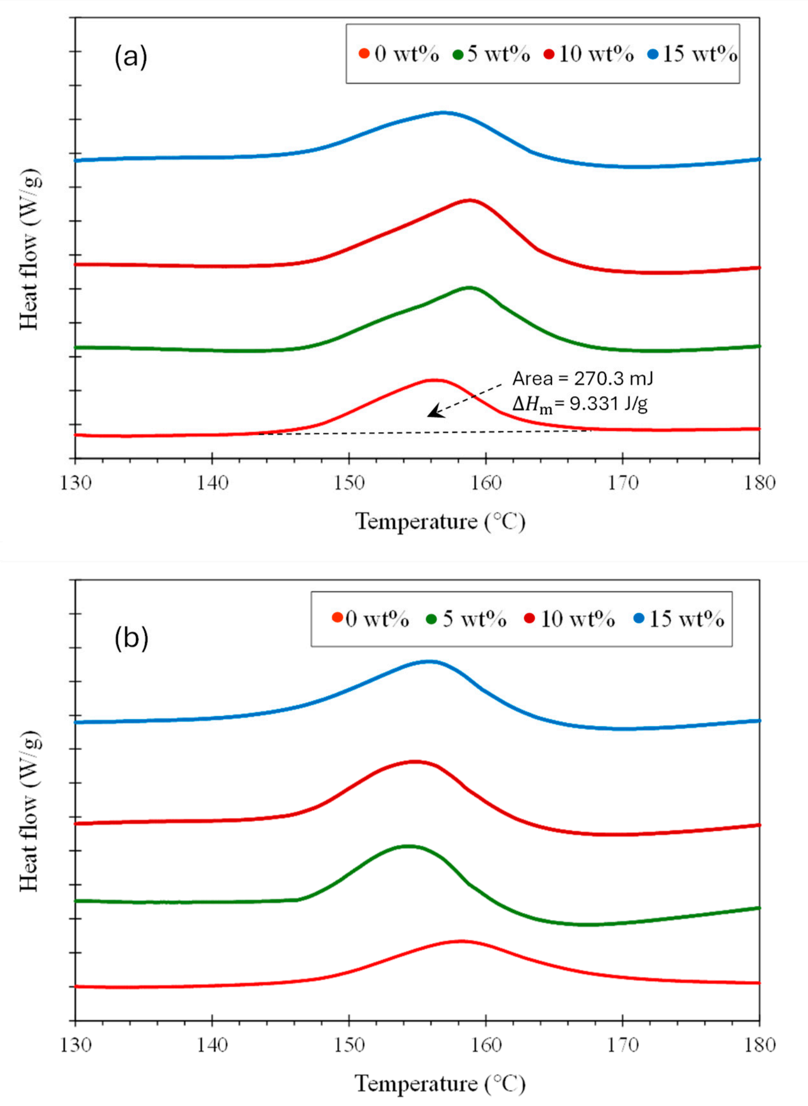

Figure S1. DSC chromatogram. The area under the curve of the peak was used to calculate the ΔH_m, the heat of fusion (J/g), which is based on the area under the curve of heat flow versus time and obtained by the operating software, Pyris 13 (Perkin-Elmer, USA). An example is shown for compression-molded 0 wt% sample. Data were vertically shifted to avoid confusion. Each division on Y-axis is 0.1 W/g. (a) compression-molded samples, (b) 3-D printed samples.
